# Neuropsychiatric Disorders in Chronic Kidney Disease

**DOI:** 10.3389/fphar.2019.00932

**Published:** 2019-08-16

**Authors:** Ana Cristina Simões e Silva, Aline Silva Miranda, Natalia Pessoa Rocha, Antônio Lúcio Teixeira

**Affiliations:** ^1^Interdisciplinary Laboratory of Medical Investigation, Faculty of Medicine, UFMG, Belo Horizonte, Brazil; ^2^Laboratory of Neurobiology, Department of Morphology, Institute of Biological Sciences, UFMG, Houston, Brazil; ^3^Neuropsychiatry Program, Department of Psychiatry and Behavioral Sciences, McGovern Medical School, University of Texas Health Science Center at Houston, Houston, TX, United States

**Keywords:** chronic kidney disease, neuropsychiatric disorders, cognition, cerebrovascular disease, anxiety, depression

## Abstract

Neuropsychiatric conditions including depression, anxiety disorders, and cognitive impairment are prevalent in patients with chronic kidney disease (CKD). These conditions often make worse the quality of life and also lead to longer hospitalizations and higher mortality. Over the past decades, some hypotheses have tried to explain the connection between CKD and neuropsychiatric disorders. The most common hypothesis is based on the occurrence of cerebrovascular disease and accumulated uremic toxins in adult patients with CKD. However, the lack of a direct association between known vascular risk factors (e.g., diabetes and hypertension) with CKD-related cognitive deficits suggests that other mechanisms may also play a role in the pathophysiology shared by renal and neuropsychiatric diseases. This hypothesis is corroborated by the occurrence of neuropsychiatric comorbidities in pediatric patients with CKD preceding vascular damage, and the inconsistent findings on neuroprotective effects of antihypertensives. The aim of this narrative review was to summarize clinical evidence and potential mechanisms that links CKD and brain disorders, specifically in regard to cognitive impairment, anxiety, and depression.

## Introduction

Several studies support the association between decreased renal function and cognitive impairement (Kurella et al., 2006; Yaffe et al., 2010; Kurella Tamura et al., 2011; Da Silva et al., 2014). For a decrease of 15 ml/min/1.73 m^2^ in glomerular filtration rate (GFR), there is an estimated decline in cognitive function similar to that of a 3-year aging (Buchman et al., 2009). Accordingly, chronic kidney disease (CKD) is an established independent risk factor for cognitive decline (Etgen et al., 2012). Psychiatric disorders are also very common in patients with CKD (Kimmel et al., 1998; Cohen et al., 2007; De Sousa, 2008; Stasiak et al., 2014). Hospitalizations due to psychiatric disorders (particularly depression, anxiety, and substance abuse) are 1.5 to 3 times more common among patients with CKD than individuals with other chronic diseases (Kimmel et al., 1998). In addition, cognitive impairment and psychiatric disorders can be leading factors of poor quality of life in CKD patients (Radic et al., 2010; Moreira et al., 2015).

Cognitive impairment has been associated with the stage of CKD, being particularly high—up to 60%—in patients undergoing hemodialysis (Murray et al., 2006; Kurella Tamura et al., 2011). The mechanisms underlying this cognitive impairment are not completely elucidated. Direct effects of uremic toxins can cause cognitive decline. However, the cognitive impairment persists despite adequate dialysis prescription, thus concluding that other factors may contribute to brain dysfunction (Radic et al., 2010). Cerebral hemodynamics dysfunction may also play a role in the pathogenesis of cognitive impairment in CKD (Skinner et al., 2005). Old age, depression, and white matter injury have also been linked to both cognitive impairment and changes in cerebral vasomotor reactivity (Da Matta et al., 2014).

Depression is the most frequently reported psychiatric condition in CKD patients, especially in those at end-stage renal disease (ESRD) (Palmer et al., 2013). The prevalence of depression among patients with CKD can be as high as 100%, depending on the diagnosis criteria and the studied population. The prevalence of depression and the risk of hospitalization due to psychiatric disturbances are higher in patients on dialysis in comparison with pre-dialysis and post-transplant patients (Palmer et al., 2013).

The neuropsychiatric manifestations in CKD patients impose unique diagnostic and therapeutic challenges. In this scenario, the aim of this narrative review was to summarize clinical evidence and potential mechanisms that links CKD and brain disorders, specifically regarding cognitive impairment, anxiety, and depression.

## Brain-Renal Axis: An Evolving Concept

Accumulating evidence has shown high prevalence of neuropsychiatric disorders, mainly cognitive decline, depression, and anxiety in CKD patients (Bugnicourt et al., 2013; Miranda et al., 2017). Indeed, the CKD-related neuropsychiatric conditions have been independently associated with poor clinical outcomes, including decrease in health-related quality of life, longer hospitalization, and higher risk for mortality (Lee et al., 2013).

A rationale for neuropsychiatric disorders secondary to kidney damage, known as the “vascular theory,” relies on the hemodynamic similarities between the brain and the kidneys (Mogi and Horiuchi, 2011). Similar anatomical and functional regulations of the microvasculature in renal and brain tissues may account for susceptibility of both organs to vascular damage and to traditional cardiovascular risk factors, including aging, obesity, diabetes, hypertension, dyslipidemia, and smoking (Toyoda and Ninomiya, 2014; Lau et al., 2017). Importantly, CKD have been regarded as a nontraditional risk factor for stroke, sleep apnea, chronic inflammation, and malnutrition (Bang et al., 2015).

Because of the vascular and hemodynamic similarities between the brain and the kidneys, it is reasonable to speculate that the microvascular damage in the kidney mirrors that in the brain. In this regard, not only CKD has been recognized as a risk factor for stroke and vascular dementia, but also it can be associated with subclinical cerebrovascular diseases. Accordingly, reduced kidney function has been independently associated with worse microstructural integrity of brain white matter, as evaluated by diffusion tensor imaging (DTI) magnetic resonance imaging (MRI) (Sedaghat et al., 2015). Also, albuminuria has been associated with larger white matter volume and decreased estimated GFR with higher cerebral blood flow in nondiabetic hypertensive adults (Tamura et al., 2016). Although subclinical cerebrovascular damage in CKD can be easily detected by MRI, this is not performed routinely in clinical practice. In addition, studies about this issue are still scarce. It is important to understand the mechanisms shared by renal impairment and brain dysfunction in order to minimize the risk for future neuropsychiatric conditions due to CKD.

Despite the known association between renal damage and neuropsychiatric conditions, direct evidence linking CKD to brain damage is still missing (Lu et al., 2015). Moreover, the vascular theory is not able to fully explain CKD-related central nervous system (CNS) dysfunction, as indicated by: (i) lack of direct association between known vascular risk factors, such as diabetes and hypertension and cognitive deficits secondary to CKD; (ii) occurrence of neuropsychiatric disorders in pediatric patients with CKD preceding vascular damage; (iii) inconsistent findings regarding potential neuroprotective effects of antihypertensive drugs against cognitive decline in CKD (Seliger et al., 2004; Duron and Hanon, 2010; Moreira et al., 2015). In this context, alternative hypotheses have proposed additional mechanisms in the kidney–brain communication, including inflammation, oxidative stress, and renin–angiotensin system (RAS) (Miranda et al., 2017). It is worth highlighting that the cross-talk between brain and kidney seems to be bidirectional since CNS conditions, like migraine and traumatic brain injury, are also independent risk factors for CKD (Weng et al., 2017; Wu et al., 2017).

Inflammation is a common feature in brain and kidney lesions, being quite reasonable to assume that inflammatory mediators may facilitate the kidney–brain cross-talk. The well-recognized role of cytokines in mediating peripheral and CNS communication reinforces this hypothesis (Lu et al., 2015). For instance, patients with CKD undergoing hemodialysis exhibit elevated serum concentrations of the chemokine MCP-1/CCL2, a protein chemoattractant for monocytes. Multiple logistic regression analysis revealed that MCP-1/CCL2 levels were significantly associated with the presence of silent cerebral infarction in this population (Uchida et al., 2012). A serum proteomic profile consisting of the inflammatory mediators IL-10 and C-reactive protein exhibited 93% accuracy in predicting mild cognitive impairment secondary to CKD (Szerlip et al., 2015).

Pre-clinical studies have also shown the involvement of inflammatory cytokines in CKD-related brain dysfunction. Increased levels of interleukin (IL)-1β, IL-6, and tumor necrosis factor (TNF) were associated with oxidative DNA damage in brain cells of rats submitted to subtotal nephrectomy (Hirotsu et al., 2011). Accordingly, increased expression of NF-κB and TNF in the hippocampus and frontal cortex were associated with aversive memory and attention impairments in subtotal nephrectomized rats at 4 months after 5/6 renal mass removal (Degaspari et al., 2015).

Oxidative stress has been associated with both brain and kidney dysfunctions. The administration of antioxidant drugs significantly prevents cognitive and behavioral alterations in experimental models of CKD, indicating a potential role for oxidative stress in the interactions between kidney and brain (Deng et al., 2001; Fujisaki et al., 2014). A significant increase of nitrotyrosine—a reactive and cytotoxic product generated by the interaction of nitric oxide (NO) and reactive oxygen species (ROS)—has been found in the brain cortex of nephrectomized rats at 6 weeks after 5/6 nephrectomy. Importantly, a protective effect was obtained with the administration of a potent antioxidant, lazaroid. This antioxidant was able to normalize the plasma levels of the lipid peroxidation product and malondialdehyde and to decrease the concentration of nitrotyrosine in the cerebral cortex of nephrectomized rats (Deng et al., 2001). The administration of tempol, another antioxidant compound, prevented spatial working memory impairment in a murine model of CKD. The protective effect of tempol was associated with inhibition of oxidative DNA damage in the hippocampus independently of renal function improvement (Fujisaki et al., 2014). More recently, in an experimental study with CKD induced by 4 weeks of adenine-rich diet, animals developed depressive-like behavior, locomotor alterations, and cognitive decline. In parallel with these behavioral and cognitive changes, animals also had decreased catalase and increased superoxide dismutase activities, elevated lipid peroxidation, and enhanced NOS-active neurons and dysfunction of mitochondrial complexes in key areas like striatum, substantia nigra, cortex, and hippocampus (Mazumder et al., 2019). Altogether, these experimental studies support the involvement of oxidative stress in neuropsychiatric disorders secondary to CKD.

The potential role of the RAS in kidney–brain crosstalk has also been investigated. The treatment with both ACE inhibitors and AT_1_ receptor antagonists exerted neuroprotective effects against the development of neurodegenerative diseases, besides exerting renoprotection (Kaur et al., 2015; Villapol and Saavedra, 2015). O’Caoimh et al. (2014) reported that patients with Alzheimer’s disease receiving ACE inhibitors have a reduced rate of functional decline. ACE inhibitors also exerted neuroprotective actions in a rat model of Parkinson’s disease (Lopez-Real et al., 2005). Treatment with captopril reduced oxidative stress and protected dopaminergic neurons in a 6-hydroxydopamine rat model of Parkinson’s disease (Lopez-Real et al., 2005). Similar results were obtained with the administration of AT_1_ receptor antagonists in patients and in experimental models of Alzheimer’s disease, Parkinson’s disease, stroke, traumatic brain injury, and spinal cord injury (Villapol and Saavedra, 2015). Our research group has investigated the profile of RAS molecules in the blood and/or cerebrospinal fluid (CSF) of patients with different neuropsychiatric conditions, including Parkinson’s disease (Rocha et al., 2016), Alzheimer’s disease (Rocha et al., 2018), and schizophrenia (Mohite et al., 2018). In patients with Parkinson’s disease, lower circulating levels of angiotensin II (Ang II) and Ang-(1–7) were associated with increased severity of depressive symptoms (Rocha et al., 2016). Patients with Alzheimer’s disease had decreased levels of ACE when compared with controls, and there was a significant positive correlation between ACE and amyloid-β_42_ concentrations in the CSF of patients (Rocha et al., 2018). Patients with schizophrenia exhibited reduced circulating levels of ACE in comparison to controls (Mohite et al., 2018).

The treatment with ACE inhibitors and AT1 receptor antagonists also exerted neuroprotection in experimental models of ESRD. 6 weeks after 5/6 nephrectomy, rats treated with the ACE inhibitor captopril decreased oxidative stress, ROS–NO interaction, and tyrosine nitration production in the cerebral cortex (Deng et al., 2001). Also, mice submitted to 5/6 nephrectomy and treated for 8 weeks with 0.5 mg/kg/day of telmisartan, an AT1 receptor blocker, improved spatial memory impairment as measured by the radial arm water maze test. The prevention of cognitive decline was associated with reduction in brain oxidative DNA damage and lipid peroxidation, supporting the hypothesis that increased action of Ang II in the CNS may underlie CKD-associated neuropsychiatric disorders (Haruyama et al., 2014).

Based on the counter-regulatory role played by the RAS axis formed by the enzyme ACE2, angiotensin-(1–7) (Ang-[1–7]), and the Mas receptor, usually opposing the actions of the ACE–Ang II–AT_1_ axis, it is expected that treatment with Ang-(1–7) and/or ACE2 activators might also lead to neuroprotection. Wang et al., 2016 showed that mice with genetic deletion of ACE2 displayed impaired cognition probably due to reduced levels of BDNF mRNA and protein in the hippocampus and increased oxidative stress. Additionally, intracerebroventricular infusion of Ang-(1–7) improves cognitive and memory decline in an experimental model of Alzheimer’s disease (Uekawa et al., 2016). Intracerebroventricular infusion of Ang-(1–7) was also able to reverse anxiety- and depression-like behaviors of hypertensive transgenic (mRen2) rats with RAS overactivity (Almeida-Santos et al., 2016). However, only few studies evaluated the ACE2-Ang-(1–7)-Mas receptor axis in neuropsychiatric disorders. Future studies are warranted to clarify the mechanisms and brain areas mediating the neuroprotective effects of ACE2-Ang-(1–7)-Mas receptor axis. It also remains to be investigated the RAS axes in cognitive impairment, anxiety, and depression related to CKD.

**Figure 1:**
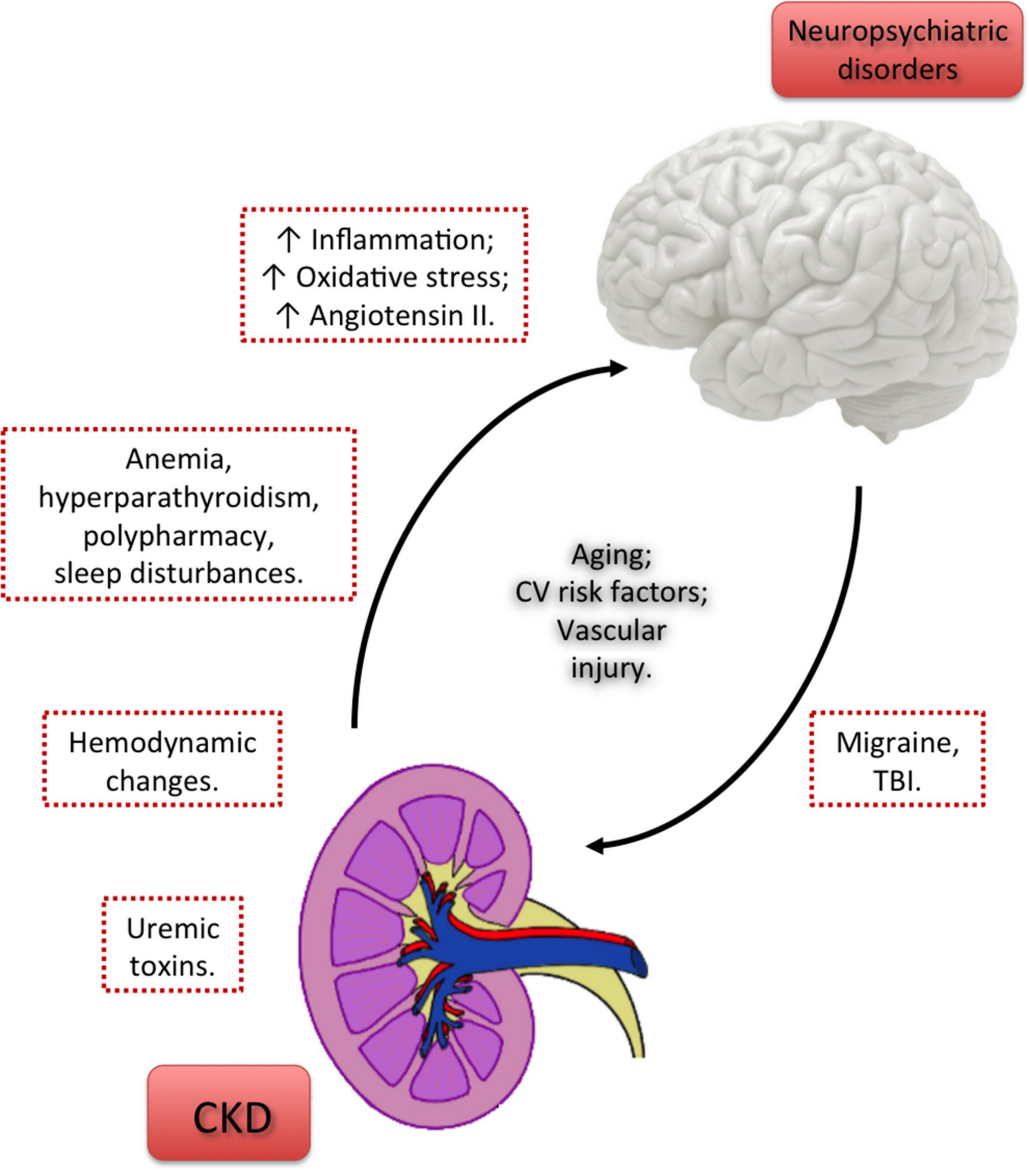
Factors linking chronic kidney disease and neuropsychiatric disorders. Uremic toxins released as a result of CKD directly contribute to brain damage and the consequent cognitive decline and psychiatric disorders. However, the persistence of neuropsychiatric conditions despite adequate dialysis prescription points out that other factors may probably contribute to brain dysfunction. Hemodynamic changes, anemia, hyperparathyroidism, polypharmacy, and sleep disturbances due to CKD may represent a link between CKD and neuropsychiatric disorders. Other factors, shared by kidney and brain tissue injuries, as the increase in the levels of inflammatory molecules, reactive oxygen species and Angiotensin II may also contribute to kidney-to-brain interactions and, consequently, to neuropsychiatric comorbidities in CKD patients. The cross-talk between brain and kidney seems to be bidirectional, since central nervous system diseases, like migraine and TBI, are independent risk factors for CKD. Aging, CV risk factors, and vascular injury represent risk factors shared by CKD and neuropsychiatric disorders, notably cognitive impairment.CKD, chronic kidney disease; CV, cardiovascular; TBI, traumatic brain injury.

**Figure 1** shows factors linking CKD and neuropsychiatric disorders.

## Cognitive Impairment in CKD

Cognitive impairment is defined by the decline in one or more cognitive domains, as perceived by the individual or a reliable informant and/or observed and documented by a clinician. There must be a clear decline from a previously higher cognitive level, and the impairment must not be better explained by another psychiatric condition or delirium.

Dementia (or major neurocognitive disorder) is diagnosed when the cognitive impairment is severe enough to interfere with independence in everyday activities (Americanpsychiatricassociation, 2013; Hugo and Ganguli, 2014). The number of people living with dementia increases exponentially with increasing age. In 2010, the number of people living with dementia worldwide has been estimated to be 35.6 million. By 2050, this number is expected to reach 115.4 million people. Dementia is an important cause of death, hospitalizations, skilled nursing facility admissions, and home health care burden (Hugo and Ganguli, 2014). The global annual costs of US$818 billion associated with dementia are expected to increase significantly in the near future (Shah et al., 2016).

The main risk factors for cognitive impairment and dementia are increasing age, lower educational level, cardiovascular disease, stroke, head injury, lifestyle habits such as smoking and heavy alcohol consumption, and psychiatric disorders notably late-life depression and anxiety (Hugo and Ganguli, 2014). The identification of risk factors and the understanding of the impact and interactions of non-modifiable (e.g., sex, genetics, age) and modifiable risk factors (e.g., educational level, habits) for dementia have been identified as one of the research priorities to reduce the global burden of dementia (Shah et al., 2016). Addressing the modifiable risk factors for cognitive impairment and dementia would significantly benefit millions of patients, their families, and society.

CKD is an independent risk factor for cognitive impairment and dementia (Etgen et al., 2012). The prevalence of cognitive impairment in individuals with kidney failure has been reported to be around 30 to 60% (Madero et al., 2008). Cognitive dysfunction can potentially affect the CKD patients’ ability to make decisions and to understand the complex treatment, including fluid and dietary restrictions (Dahbour et al., 2009; Sorensen et al., 2012). Additionally, patients with cognitive impairment present an increased risk of hospitalization, mortality, and poor quality of life (O’lone et al., 2016).

The association between CKD and cognitive impairment can be explained by several factors. First, patients with CKD have a higher prevalence of cerebrovascular disease and cardiovascular risk factors than the general population. Traditional vascular risk factors, i.e., hypertension, hypercholesterolemia, diabetes mellitus, smoking, and cardiovascular disease, are strongly associated with CKD, cerebrovascular disease, and dementia. Second, nontraditional vascular risk factors such as hyperhomocysteinemia, hemostatic abnormalities, and hypercoagulable states are frequently detected in CKD patients and have been associated with cognitive impairment. Third, increased oxidative stress and inflammation due to CKD are also associated with cognitive impairment and dementia. Finally, nonvascular risk factors such as anemia, hyperparathyroidism, polypharmacy, sleep disorders, and depression may represent an additional link between CKD and cognitive decline (Madero et al., 2008). Furthermore, dialysis patients undergo hypoxemia, large fluid and osmolar shifts, fluctuating uremic toxin titers, and a proinflammatory state. All these factors can potentially affect cognitive function. In fact, patients under hemodialysis have worse cognitive performance when compared to the general population, particularly in the orientation, attention, and executive function domains (O’lone et al., 2016).

A recent study indicated that for every 10 ml decrease in the estimated GFR below 60  ml/min/1.73 m^2^, there is an 11% increase in the risk of cognitive impairment (Tian et al., 2019). A meta-analysis of cross-sectional and longitudinal studies comprising 54,779 participants corroborated these findings. Not only the study concluded that CKD is significantly associated with cognitive decline, but this association was independent of the CKD stage and was stronger in the group with moderate-to-severe CKD compared with mild-to-moderate CKD (Etgen et al., 2012).

There are several studies investigating cognitive performance across the CKD spectrum, i.e., pre-dialysis CKD patients, patients on renal replacement therapy (hemodialysis or peritoneal dialysis), and transplant recipients for a systematic review, see (Vanderlinden et al., 2019). A recent meta-analysis found that ESRD patients submitted to different modalities of renal replacement therapy have distinct cognitive deficits. Both pre-dialysis and patients on hemodialysis exhibited worse global cognition performance in comparison with non-CKD controls, as demonstrated by the significantly lower scores on the Mini-Mental State Examination. Also, patients on peritoneal dialysis or hemodialysis had worse attention/working memory performance, as evaluated by the Trail Making Test-A, than non-CKD controls (Vanderlinden et al., 2019).

Hemodialysis and peritoneal dialysis are equivalent in terms of survival (Yeates et al., 2012), and both dialysis modalities are associated with high prevalence (60–70%) of moderate to severe cognitive impairment (Kalirao et al., 2011). However, studies have reported better cognitive outcomes in patients on peritoneal dialysis compared with patients on hemodialysis (Tilki et al., 2004). In uremic patients, although the hemodialysis was able to restore a normal cognitive function, this effect was observed only transiently in the post-dialytic phase. Differently, peritoneal dialysis preserved cognitive function steadily close to normal (Buoncristiani et al., 1993). In addition, peritoneal dialysis has been reported to be more effective than hemodialysis in reversing uremic encephalopathy (Wolcott et al., 1988), and the risk of dementia for patients who started on peritoneal dialysis is lower compared with those who started on hemodialysis (Wolfgram et al., 2015). These results were confirmed by a recent systematic review and meta-analysis, which concluded that peritoneal dialysis is better in preserving the cognitive functions and is associated with a lower risk of dementia in comparison with hemodialysis (Tian et al., 2019).

Taken together, patients with CKD exhibit worse cognitive performance than the general population. The clinical phenotype and severity of cognitive impairment may depend on the renal replacement therapy, with peritoneal dialysis showing better outcomes than hemodialysis. There are potentially biased studies that did not control for educational level and other confounding variables, besides a high heterogeneity of results, mainly due to the large variety of tests used to assess cognition. Cognitive deficits in specific domains must be better investigated and considered for disease management. Accordingly, larger studies with more careful design, including comprehensive neuropsychological and behavioral phenotyping, are needed to draw more definite conclusions. Noteworthy, aging is a common risk factor for both cognitive impairment and CKD (Bowe et al., 2018). Given that the incidence of CKD is increasing, particularly in the elderly, recognizing and understanding cognitive dysfunction in CKD patients have become a research priority as well.

It is worth mentioning that the relationship between cognitive impairment and reduced renal function seems to be bidirectional. For instance, a case-control study has shown that people with Alzheimer’s disease have greater renal impairment than controls, even after adjustment for age, diastolic blood pressure, apolipoprotein E (APOE) ɛ4 genotype and education level (Kerr et al., 2009). Also, the reduced renal function can worsen clinical symptoms in patients with cognitive impairment. For instance, it has been reported that psychotic symptoms are associated with poorer renal function in people with mild cognitive impairment and Alzheimer’s disease (Kunschmann et al., 2017).

## Depression and Anxiety in CKD

Psychiatric conditions, especially depression and anxiety, are commonly found in CKD patients. Psychiatric disorders in CKD population have been associated with significant decline in overall quality of life, rapid progression to ESRD, as well as higher risk of hospitalization and death (Hedayati et al., 2010; Cukor et al., 2012; Tsai et al., 2012; Chiang et al., 2015).

### Depression

Depression is highly prevalent in patients with CKD. A systematic review and meta-analysis that analyzed 216 studies involving 55,982 patients with CKD or ESRD showed a prevalence of 26.5% of depressive symptoms in CKD patients when evaluated by screening questionnaires, and of 21.4% of clinically significant depression when evaluated by clinical interview (Palmer et al., 2013). The prevalence of depression in CKD patients is three to four times higher compared with the general population and two to three times higher compared to other chronic diseases including diabetes, coronary artery disease, and chronic obstructive pulmonary disease (Waraich et al., 2004; Katon, 2011; Pratt and Brody, 2014). Accordingly, the rate of antidepressant prescription is nearly 1.5 times higher in CKD patients than in the general population (Iwagami et al., 2017).

Demographic, socioeconomic, and clinical risk factors including younger age, female sex, Black race, Hispanic ethnicity, lower education, lower family income, unemployment, hypertension, smoking status, and diabetes have been associated with depression secondary to CKD (Kop et al., 2011; Fischer et al., 2012; Tsai et al., 2012). Based on the fact that these risk factors seem to be more frequently in CKD patients compared with the general population, they may explain, at least in part, the higher prevalence of depressive symptoms in the CKD population (Nicholas et al., 2015). CKD also influences the emotional state of the patients due to of several stressors, including adjustments to a strict dietary and fluid restriction, and occurrence of pain and fatigue (Kimmel, 2002; Davison and Jhangri, 2010).

It has been reported that depression in CKD might be related with poor clinical outcomes, which include hospitalization, kidney function decline, progression to ESRD, and mortality (Hedayati et al., 2010; Tsai et al., 2012; Chiang et al., 2015). A prospective study with a mean follow-up of 2 years evaluated the association of depression and renal function decline in 568 patients with CKD. Individuals with depressive symptoms (160 subjects) presented a faster decline in estimated GFR and were 1.7 times more likely to progress to ESRD or death than those without depression (Tsai et al., 2012). Decline in GFR was also reported in CKD patients with elevated depression scores in the Beck Depression Inventory (BDI) in a 6-month follow-up study. Notably, depressive symptoms were associated with adverse psychosocial outcomes such as poor quality of life, inferior social support, and worse community integration (Cukor et al., 2012). A negative correlation was found between quality of life measures and depression, as measured by the Hospital Anxiety and Depression Scale in pre-dialysis CKD patients. This finding reinforces the concept that detection and adequate treatment of depressive symptoms might improve the quality of life of these patients (Lee et al., 2013). A cross-sectional study with 152 CKD patients reported a prevalence of depressive symptoms in 27% of the subjects who were starting renal replacement therapy by hemodialysis or peritoneal dialysis. Depressive symptoms affected both physical and emotional components of quality of life as measured by the Kidney Disease Quality of Life Short Form (Rebollo Rubio et al., 2017). Similar findings were reported in a cross-sectional study with 335 ESRD patients on hemodialysis (Li et al., 2016). Poor quality of life and lower resilience were also associated with depression in pediatric (age range from 9 to 18 years) patients at stages 1 to 4 of CKD (Moreira et al., 2015).

Depression is an independent risk factor for hospitalization and death in both patients receiving dialysis or at pre-dialysis stages of CKD (stages 1–4) (Lopes et al., 2002; Hedayati et al., 2005; Hedayati et al., 2008; Hedayati et al., 2010). In 1-year follow-up study with 267 CKD patients at stages 2–5 not under dialysis, major depression was observed in 56 (21%) patients and, at the end of 1 year, the diagnosis of depression at baseline independently predicted progression to dialysis and hospitalization. The poor outcomes in CKD patients with depressive symptoms were not related with the presence of comorbidities or kidney disease severity (Hedayati et al., 2010). A more recent cohort study followed pre-dialysis CKD patients for 3 years to investigate whether depression is an independent risk factor for initiation of dialysis and for mortality. A total of 262 CKD patients was enrolled in the study, with 56 (21.4%) presenting clinically meaningful depressive symptoms at baseline. In line with the previous report (Hedayati et al., 2010), the presence of depressive symptoms at baseline independently predicted the risk of initiation of dialysis and mortality (Chiang et al., 2015). Another prospective study showed that patients with more depressive symptoms at baseline had higher risk of hospitalization and death due to cardiovascular complications (Fischer et al., 2011).

The presence of depression at the time of dialysis onset is also an independent predictor of lower survival rates, greater frequency of dialysis withdrawal, higher risk of hospitalization, and longer hospitalization (Chilcot et al., 2011; Lacson et al., 2012; Lacson et al., 2014). A longitudinal study investigated for 2 years the occurrence of depressive symptoms and frailty in 771 patients on dialysis and whether these conditions were independently associated with mortality. At baseline, 13.1% of individuals presented depressive symptoms based on the Center for Epidemiologic Studies Depression scale, 21.8% had frailty, and 10.0% met criteria for both. After 2 years of follow-up, 26.6% of CKD patients had frailty, and 12.7% exhibited depressive symptoms, and depressive symptoms and frailty were independent predictors of mortality (Sy et al., 2019).

Despite its high prevalence and significant clinical and socioeconomic burden, depression seems to be undertreated in patients with CKD. A large cross-sectional involving 1,099 adults with CKD stages 3 to 4 who had depressive symptoms as defined by the score of 11 or higher in the BDI revealed that only 31% of the patients reported the prescription of antidepressants (Fischer et al., 2012). The low prescription rate of antidepressants among the CKD population may rely on the fact that these drugs are highly protein-bound and metabolized by the liver, making them unlikely to be removed by dialysis, raising medical concerns with their prescription (Hedayati et al., 2012). The first-line antidepressants for CKD patients are the selective serotonin re-uptake inhibitors. However, few studies have investigated the safety and efficacy of these medications in CKD patients, and most of them had significant limitations including small sample sizes, lack of control group, and selection and drop-out bias (Nagler et al., 2012; Palmer et al., 2016). Treatment of CKD-related depressive symptoms must also include non-pharmacological strategies like psychotherapy (e.g., cognitive-behavioral therapy), exercise training programs, and social support (Symister and Friend, 2003; Duarte et al., 2009; Ouzouni et al., 2009; Kouidi et al., 2010). A randomized trial with 85 patients on hemodialysis and presenting with depressive symptoms obtained a significant improvement in the BDI score following 12-week sessions of cognitive-behavioral therapy (Duarte et al., 2009). Exercise training programs can reduce depressive symptoms in dialysis patients, but the improvement depends on at least 6 months of intervention (Ouzouni et al., 2009; Kouidi et al., 2010). Whether CKD patients with pre-existing depression would benefit from physical activity intervention still deserves investigation, as these individuals may lack the motivation to engage in exercise programs. Finally, social support is also a promising strategy to decrease depressive symptoms, specifically by increasing optimism and self-esteem (Symister and Friend, 2003). Although non-pharmacological approaches play a definite role for the management of CKD-related depression, several factors including lack of patients willingness to follow recommendations and limited availability of those non-pharmacological strategies in CKD clinics or dialysis centers hamper their integration in the clinical practice (Green et al., 2012; Weisbord et al., 2013; Hedayati et al., 2016). In this regard, a clinical trial was recently conducted in patients receiving hemodialysis in order to assess: (i) the effect of an engagement interview on patients’ willingness to accept treatment for depression and (ii) the efficacy of cognitive-behavioral therapy in comparison with sertraline for treating depression. The engagement interview did not affect patients’ acceptance of treatment for depression (which was 64–66%). Both cognitive-behavioral therapy and sertraline improved depressive symptoms and other secondary outcomes such as energy/vitality and sleep quality. The outcome scores were modestly better for the sertraline group, which also presented more frequent adverse events in comparison with the cognitive-behavioral therapy group (Mehrotra et al., 2019).

### Anxiety

Anxiety is also a common psychiatric condition in patients with CKD, although this condition has been substantially lesser studied than depression. A longitudinal study conducted with 50 CKD patients on hemodialysis revealed symptoms of anxiety in 45.7% of them, as assessed by the Hospital Anxiety and Depression Scale (HADS). After 16 months of follow-up, a significant portion of these CKD patients (30%) remained with anxiety symptoms (Cukor et al., 2008). A 3-year follow-up study showed that 31 among 100 patients with pre-dialysis CKD exhibited anxiety symptoms evaluated by the Beck Anxiety Inventory (Loosman et al., 2015).

High prevalence of anxiety in CKD population has been reported in cross-sectional studies as well. In a study involving 208 pre-dialysis CKD patients, the frequency of anxiety, as assessed by the HADS, was found to be 24.8% in patients at CKD stage 3, 29.9% in patients at stage 4, and 34.3% in patients at stage 5 (Lee et al., 2013). No significant differences were detected in the frequency of anxiety symptoms according to CKD stages (Lee et al., 2013). In a cross-sectional study including 155 patients undergoing hemodialysis for at least 6 months, anxiety symptoms evaluated by Beck Anxiety Inventory were found in 53%, being moderate to severe symptoms in 28% of patients (Feroze et al., 2012). The frequency of anxiety symptoms was not influenced by the method of renal replacement therapy. Similar scores were obtained in BAI and HADS for 128 patients on hemodialysis in comparison with 27 on peritoneal dialysis (Stasiak et al., 2014). Additionally, comparable frequencies of anxiety symptoms were also found in CKD patients before (21.6% of a total of 101 individuals) and after kidney transplantation (25% of a total of 151 patients), as assessed by the HADS (Muller et al., 2015). A more recent study conducted with 152 CKD patients starting renal replacement therapy identified anxiety symptoms in 26.6% of the patients. Taken together, these studies showed that anxiety symptoms are at least two times higher in CKD patients in comparison to general population (Kessler et al., 2005; Feroze et al., 2012).

Anxiety symptoms may be associated with poor clinical and psychological outcomes like poor health–related quality of life, hospitalization, and mortality. Regarding the health-related quality of life, prospective and cross-sectional studies have shown lower scores of quality of life related to the stage of CKD. In these studies, the Kidney Disease Quality of Life questionnaire and the Medical Outcomes Survey 36-item Short Form (SF-36) were employed to evaluate the health-related quality of life in CKD patients, and the most pronounced impairments were in physical function and physical scales (Molsted et al., 2007; Mujais et al., 2009; Pagels et al., 2012; Lee et al., 2013; Li et al., 2016). Anxiety symptoms were independently associated with impairment in physical and emotional components of health-related quality of life in pre-dialysis CKD patients at the start of dialysis and in patients under hemodialysis (Lee et al., 2013; Kang et al., 2015; Rebollo Rubio et al., 2017). It is worth noticing that lower scores for health-related quality of life domains have been associated with higher risks for ESRD and for all-cause mortality in CKD patients (Tsai et al., 2010). Anxiety symptoms also seem to be an independent risk factor for hospitalization and mortality amongst CKD patients. A 3-year prospective cohort study including 100 pre-dialysis CKD patients showed that anxiety symptoms were associated with adverse clinical outcomes such as death, initiation of dialysis, or hospitalization (Loosman et al., 2015). A recent prospective cohort study showed that anxiety symptoms were independently associated with increased risk for mortality and days of hospitalization (Schouten et al., 2019).

Few studies have investigated therapeutic strategies for CKD-associated anxiety symptoms. Although benzodiazepines are often prescribed for the treatment of acute episodes of anxiety, their use should be avoided due to high rates of side effects, including drowsiness, risk of falls, and abuse liability (Cohen et al., 2004; Yeh et al., 2014). As for CKD-related depression, the first-line pharmacological strategy recommended to treat anxiety disorders in CKD patients is the selective serotonin re-uptake inhibitors (Bandelow et al., 2012). A recent study showed that a brief cognitive-behavioral intervention consisting of positive self-reinforcement, deep breathing, muscle relaxation, and cognitive restructuring decreased anxiety and depressive symptoms and improved quality of life after 4-week follow-up compared with the baseline scores. This study was conducted in ESRD patients on hemodialysis (Lerma et al., 2017). The benefits of cognitive-behavioral intervention and other non-pharmacological strategies for early stages of CKD still need to be evaluated.

## Concluding Remarks

The interactions between kidney and brain are complex and multifaceted, thus justifying the significant neuropsychiatric comorbidity observed in patients with CKD. Cognitive impairment, highly prevalent in CKD patients, may be linked, but not exclusively, to common susceptibility of brain and kidney tissues to vascular injury. Depression and anxiety, also frequently diagnosed in all stages of CKD, cannot be explained by neuronal dysfunction related to uremic state or vascular injury. Alongside psychosocial factors, other pathological mechanisms, shared by both kidney and brain tissue injuries, as inflammatory mediators, ROS and components of the RAS might contribute to cerebrorenal interactions and, consequently, to neuropsychiatric comorbidities in CKD patients.

A direct link between CKD and brain damage is still elusive. Understanding the pathophysiology of these interactions between chronic renal impairment and brain dysfunction is pivotal to prevent and/or minimize the occurrence and impact of cognitive impairment, depression, and anxiety in CKD patients.

## Author Contributions

ACSS and ALT proposed the topics and made general supervision. NPR and ASM searched for articles and wrote the first draft of the review. All authors revised the manuscript and approved the final version.

## Funding

This study was partially supported by CNPq (grants number 301037/2016-7 and 406041/2018-0) and FAPEMIG (grant number CDS - APQ-02541-17).

## Conflict of Interest Statement

The authors declare that the research was conducted in the absence of any commercial or financial relationships that could be construed as a potential conflict of interest.
